# Random walk diffusion simulations in semi-permeable layered media with varying diffusivity

**DOI:** 10.1038/s41598-022-14541-y

**Published:** 2022-06-24

**Authors:** Ignasi Alemany, Jan N. Rose, Jérôme Garnier-Brun, Andrew D. Scott, Denis J. Doorly

**Affiliations:** 1 Department of Aeronautics, Imperial College London, South Kensington Campus, London, SW7 2AZ UK; 2 Cardiovascular Magnetic Resonance Unit, Royal Brompton Hospital, Sydney Street, London, SW3 6NP UK; 3 National Heart and Lung Institute, Imperial College London, London, SW3 6LY UK; 4 Chair of Econophysics and Complex Systems, École Polytechnique, 91128 Palaiseau Cedex, France; 5 LadHyX UMR CNRS 7646, École Polytechnique, 91128 Palaiseau Cedex, France

**Keywords:** Computational biophysics, Biological physics, Biological models

## Abstract

In this paper we present random walk based solutions to diffusion in semi-permeable layered media with varying diffusivity. We propose a novel transit model for solving the interaction of random walkers with a membrane. This hybrid model is based on treating the membrane permeability and the step change in diffusion coefficient as two interactions separated by an infinitesimally small layer. By conducting an extensive analytical flux analysis, the performance of our hybrid model is compared with a commonly used membrane transit model (reference model). Numerical simulations demonstrate the limitations of the reference model in dealing with step changes in diffusivity and show the capability of the hybrid model to overcome this limitation and to offer substantial gains in computational efficiency. The suitability of both random walk transit models for the application to simulations of diffusion tensor cardiovascular magnetic resonance (DT-CMR) imaging is assessed in a histology-based domain relevant to DT-CMR. In order to demonstrate the usefulness of the new hybrid model for other possible applications, we also consider a larger range of permeabilities beyond those commonly found in biological tissues.

## Introduction

Diffusion of fluid particles within a material consisting of multiple compartments separated by semi-permeable barriers or membranes is important in a vast number of areas such as heat transfer problems^1,2^, mathematical modelling in finance^3^ or social dynamics^4^, astrophysics^5^, the study of porous media^6,7^, and magnetic resonance imaging based diffusion-weighted imaging (DWI). Magnetic resonance imaging has been a significant spur for the development of both analytical methods^8,9^ to treat diffusion in permeable layered media, as well as for the development of Monte Carlo random walk methods^10–12^ to model the process. Random walk methods are computationally attractive for the solution of diffusion in large, complex configurations and play an important role in understanding magnetic resonance–based diffusion measurements. However simulating DWI is computationally demanding and the existing random walk methods are subject to limitations in their capacity to treat semi-permeable membranes interposed between regions of different diffusion coefficients. The purpose of this work is firstly to describe an improved transit model capable of overcoming such restrictions, secondly to validate with a semi-analytical model^9^, and thirdly to investigate the resulting improvement gained in modelling DWI of biological tissue. In the remainder of the introduction, we briefly explain the relation between DWI and diffusion simulations, referring to both analytical and Monte Carlo methods, before outlining the structure of the paper.

DWI is a unique magnetic resonance imaging technique that provides measures relating to the average microscopic structure within a macroscopic imaging voxel by measuring the displacement of water molecules due to self-diffusion over a given time (the diffusion time,  $\Delta$)^13,14^. DWI-based methods are widely used in neuroscience for determining white matter pathways, for example via the primary eigenvector of the $3 \times 3$ diffusion tensor^15,16^ which aligns with the long axis of the neurons. More recently, DWI techniques have gained popularity for cardiac imaging, where they can be used to investigate the unique variations in the arrangement of heart muscle cells in space and in time as the heart contracts^17–19^.

Monte Carlo simulations are a well-established method for investigating the relationship between the properties of the microscopic structure of biological tissues and the apparent diffusivity which would be measured in DWI methods^12^. These computational simulations are becoming increasingly more realistic in terms of geometric fidelity^20–22^. Compartment models, which assume the tissue consists of a number or distribution of compartment sizes and consider the DWI signal contribution of each compartment separately, have reached a point of maturity, but are limited to tissues with no/low membrane permeability or short diffusion times. In cardiac tissue, however, diffusion times are of the order of $1\,\hbox {s}$ when employing the commonly used stimulated echo acquisition mode^23^ technique due the synchronisation with the cardiac period. As a result, the mean distance displaced by a molecule during an experiment covers multiple compartment lengths and the membranes of the typically well-mixed myocardial cells^24^ may no longer be considered impermeable.

Exchange of walkers through barriers is commonly modelled via a transit probability, where an attempt to cross the barrier is either rejected or permitted randomly based on a threshold probability. This threshold probability is dependent on some (or all) of the tissue properties so as to ensure the membrane permeability is accurately represented. Powles et al.^10^ derived a formula for the transit probability of walkers on a lattice with constant step size. Szafer et al.^11^ considered a grid of 3D rectangular cells on a regular grid, allowing for different intra- and extra-cellular diffusivities. A recent model^25,26^, which we consider the reference model for this work, attempts to improve and extend the performance of a previous published transit model^10^. This reference model requires a strict limit on the maximum time step permitted in the random walk, which may pose numerical challenges when long diffusion times are considered or a large parameter space is to be investigated.

A number of analytical approaches to the problem of diffusion also exist. Data fitting models attempt to compose the observed DWI signal as a linear combination of analytical shapes like spheres or ellipsoids for which the individual signal contribution is known^27^. This allows for the inference of cell sizes from the measured data. Originally developed for impermeable membranes, this approach was extended by Kärger et al.^28^ to account for exchange between compartments. While these models offer an easy way to explain macroscopic observations through integral quantities like the diffusion signal, they do not allow for deeper insights into the diffusion processes themselves. By reducing the problem to 1D, analytical solutions for the diffusion propagator can be found. This was first suggested by Tanner et al.^8^ to estimate the DWI signal in a system of equi-spaced parallel plates. Very recently, Moutal et al.^9^ presented a semi-analytical method to obtain the particle density distribution anywhere in a domain with arbitrary barriers.

In this work, we study 1D diffusion through semi-permeable membranes and show the numerical limitations of the previously mentioned reference transit model^25,26^. We propose a new model (hybrid model) based on treating the membrane interface and the discontinuity in diffusion coefficient as two separate probabilities. We analyse the behaviour of both transit models by comparing the fluxes through the membrane with those obtained by a semi-analytical solution. A parameter study reveals the errors in the numerical results and demonstrates that the limitations of the hybrid model are numerically less restrictive. Finally, we quantify the impact of our findings by calculating the difference in DWI signal obtained for both transit models on a realistic histology-based domain featuring a wide range of permeability values. This allows us to assess and compare the suitability of both models for simulating diffusion tensor cardiovascular magnetic resonance (DT-CMR) and other potential applications.

## Methods

**Figure 1:**
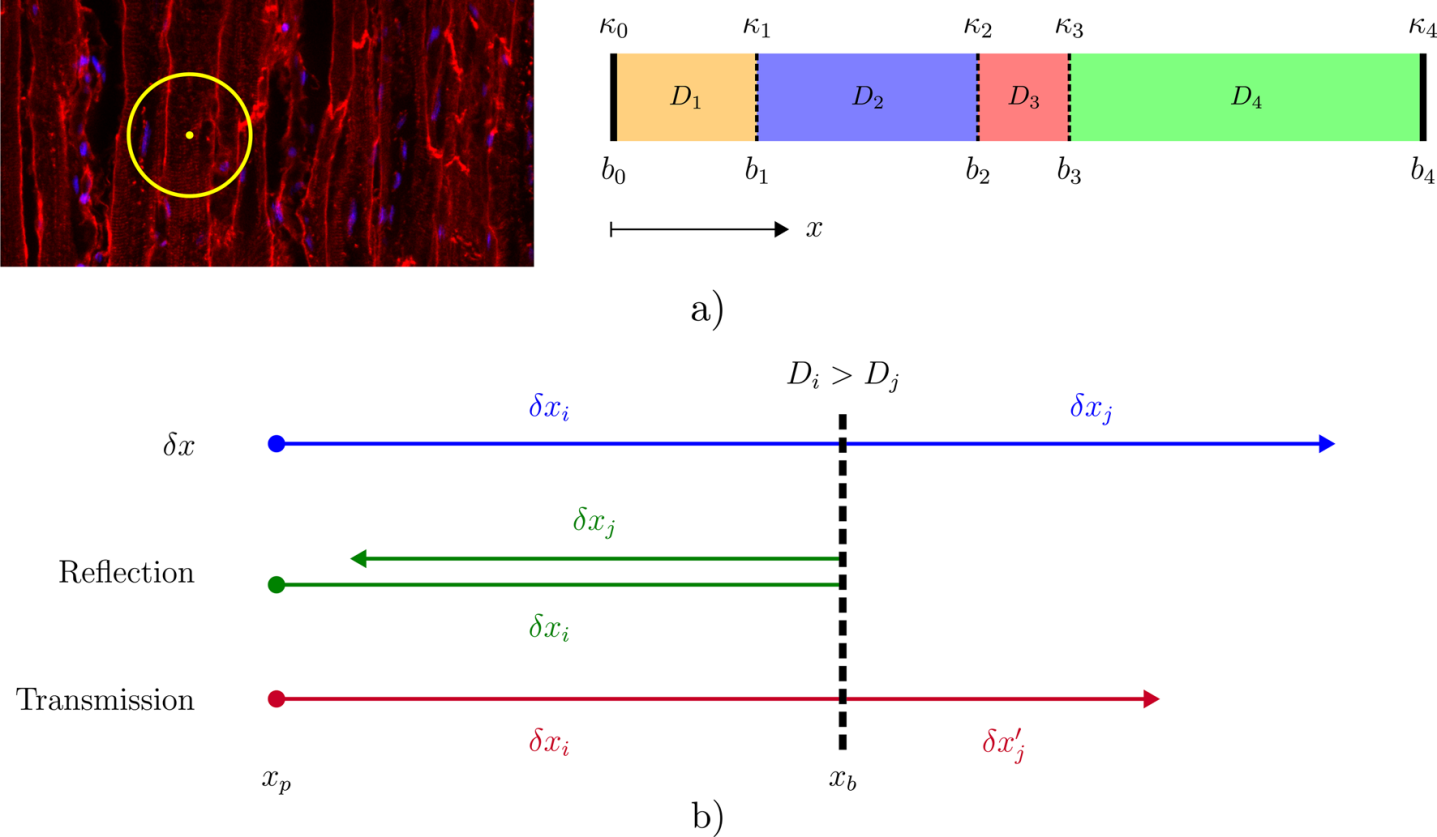
(**a**) Left: Confocal fluorescence microscopy image of cardiomyocytes running vertically. The mean diffusion distance $\Delta x \approx 30\,\upmu \hbox {m}$ over $100\,\hbox {ms}$ is indicated as the radius of the yellow circle. Right: Schematic of an example domain with $m = 4$ compartments with indices *i*. Each compartment has two barriers with their corresponding locations $x_b=b_{i}$, $x_b=b_{i+1}=b_j$ with permeabilities $\kappa _{i}$ and $\kappa _{j}$. Note that the domain ends enforce the zero-flux boundary conditions ($\kappa _{0} = \kappa _{m} = 0$). (**b**) Illustration of the behaviour of a single walker at $x_p$ performing a random step $\delta {x}$ towards a barrier at $x_b$. Initially, the step is divided into $\delta {x}_i$ and $\delta {x}_j$. Depending on the transit decision, the walker is either reflected elastically ($x = x_p + \delta {x}_i - \delta {x}_j$) or enters the new compartment with $D_j < D_i$. In the latter case, the remaining step after transit is modified to $\delta {x}'_j$ to preserve a constant net step size. Note that $|\delta {x}'_j| < |\delta {x}_j|$ when entering a region of lower diffusion coefficient (and conversely for $D_j < D_i$).

Histology images provide information on and properties of the biological tissue required for realistic DWI computational simulations. Considering that the diffusion in the vertical/longitudinal direction (parallel to the cardiomyocytes) is much less restricted than in the perpendicular direction, the 3D problem may be reduced to 2D and even 1D for many applications. Figure 1 provides an example of a histology image obtained via confocal microscopy and one simplified 1D arbitrary domain. We consider 1D domains as arrays of *m* fixed length compartments $[L_1,L_2,..,L_i,..L_m]$ each with constant diffusion coefficient $D_i$ separated by semi-permeable barriers with permeability coefficients $\kappa _{i}$. This membrane permeability $\kappa _{i}$, with units of distance over time, effectively defines the rate of exchange through the membrane between compartments *i* and $i+1$. Note that compartment $i+1$ is that immediately adjacent to *i* on the right, in the conventional left-to-right *x*-axis direction as illustrated in Fig. 1. We consider impermeable membranes at the ends of the domain, i.e. $\kappa _{0} = 0 = \kappa _{m}$, as walkers do not vanish in the biological tissue. The 1D diffusion equation for continuously variable diffusion coefficient is expressed as

1 $$\begin{aligned} \frac{\partial {U(x, t)}}{\partial {t}} = \frac{\partial {}}{\partial {x}} \Big [ D(x) \frac{\partial {U(x, t)}}{\partial {x}} \Big ] \end{aligned}$$

where *D*(*x*) is the diffusion coefficient and *U*(*x*, *t*) the particle density/probability at a position in time and space. Setting the diffusion coefficient $D_i$ as constant throughout a given compartment *i* simplifies the diffusion equation (2) to

2 $$\begin{aligned} \frac{\partial {U(x,t)}}{\partial {t}} = D_{i} \frac{\partial ^2{U(x,t)}}{\partial {x}^{2}} \end{aligned}$$

for compartments $1 \le i \le m$. This allows us to model different compartments as intra-cellular (ICS) or extra-cellular space (ECS, which may be interstitial or intravascular) by applying different reduced diffusion coefficients compared to the free diffusivity of water. At the internal interfaces $1 \le i \le m-1$ between compartments, where $\partial {D}/\partial {x} \ne 0$, the solution is defined by the boundary condition^8,10^

3 $$\begin{aligned} D_{i} \frac{\partial {U(x,t)}}{\partial {x}}\big |_L = D_{j} \frac{\partial {U(x,t)}}{\partial {x}}\big |_R = \kappa _{i} \left( \left. U \right| _R - \left. U \right| _L \right) . \end{aligned}$$

It relates the continuous diffusive fluxes $D\partial U/\partial x$ infinitesimally to the left (subscript *L*) and right (*R*) of the interface to the jump in concentration $\left. U \right| _R - \left. U \right| _L$ across the membrane with the permeability $\kappa _i$.

### Random walk transit models for permeable membranes

The solution to the compartment diffusion equation (2) may be obtained by considering an ensemble of massless, non-interacting particles performing a random walk. Below, we describe this process for a single particle/walker, which is repeated $N_p$ times per experiment.

#### A single random walker

We consider a walker with subscript *p*. The position of the walker $x_p$ inside the domain is updated during each time step $\delta {t}$ through a series of sub-steps $\delta {x}_n$ that depend on the diffusivity of the local environment:

4 $$\begin{aligned} x_p(t+\delta {t}) = x_p(t) + \sum _{n}{ \delta {x}_n }. \end{aligned}$$

At the beginning of a time step, a random step vector $\delta {x}$ is drawn with equal probability of moving in direction $+x$ or $-x$. For diffusion away from barriers, a single step $\delta {x} = \pm \sqrt{2 D_i \delta {t}}$ is performed. Interaction with barriers introduces additional sub-steps, however in this work we consider a maximum of a single barrier crossing per time step. This imposes a limit on the possible time steps, namely $\delta {t} < \delta {t}_{\text {max}}$, where $\delta {t}_{\text {max}} = \min (L_i^2/(2 D_i))$.

#### Transit probability

In Fig. 1 we illustrate the reflection and transmission of a particular walker through a barrier located at $x_b$. When the updated walker position $x_p+\delta {x}$ would lead to a crossing of a membrane, the interaction is resolved by computing a probability of transit $p_t$ and dividing the step into $\delta x_i$ and $\delta x_j$, see Fig. 1. Note that if $D_i \ne D_j$ the transit probability $p_t$ is specific to each direction ($i \rightarrow j$ or $j \rightarrow i$) and must satisfy the interface reflection^11,29^. The interaction is resolved by drawing a random number $\mathscr {U} \in [0,1)$ and comparing it to the deterministic value of $p_t$. Transit only occurs if $\mathscr {U} < p_t$, otherwise the walker is reflected elastically by $\delta x_j$. If it enters a new compartment with different diffusivity $D_j \ne D_i$, the distance moved $\delta x_j'$ in the remaining fraction of the time step needs to be adjusted to maintain a constant fractional time step^11,29^.

#### Hybrid model

A number of “transit models” have been proposed to calculate the probability of transit $p_t$ based on properties of the membrane and tissue. The aim of any such model is to accurately represent the boundary condition^8,10^ in Eq. (3) that relates the diffusive flux to the jump in particle density *U* across the semi-permeable membrane. The case of a finite membrane permeability with equal diffusivity on either side (i.e. $D_i = D_j$) is well-studied on a discrete lattice of equidistant points^10^. In the reference model^25,26^ this approach is extended to particles located at arbitrary positions $x_p$ in the vicinity of a membrane separating regions of equal diffusivity:

5 $$\begin{aligned} p_{t_{\large {ref}} , (i \rightarrow j)} = \frac{2 \kappa _{i} \delta {x}_i}{D_i + 2 \kappa _{i} \delta {x}_i}. \end{aligned}$$

Here $\delta {x}_i$ is the distance from the walker position $x_p$ to the barrier $x_b=b_i$. It can be shown (Appendix A) that a step change in diffusion coefficient ($D_i \ne D_j$) leads to an error in the model proposed by Fieremans et al.^25^. This error reduces with the time step and it is thus recommended by the authors to keep $\delta {t} < \delta {t}_{\text {max ref}}$ such that $p_{t_{\large {ref}}} \ll 1$, i.e. $p_{t_{\large {ref}}}(\delta {t}_{\text {max ref}}) < 0.01$, for the transit probability

Unfortunately, in the limiting case of infinite permeability, the reference model is inconsistent with the condition derived by Maruyama et al.^29^ for particle transit across a step change in diffusion coefficient. Thus with the motivation to create a consistent treatment and to lift the step restriction when $D_i \ne D_j$, we propose a hybrid transit model based on treating the step change in diffusion coefficient and the membrane permeability as two independent processes during the transit of a walker.

**Figure 2:**
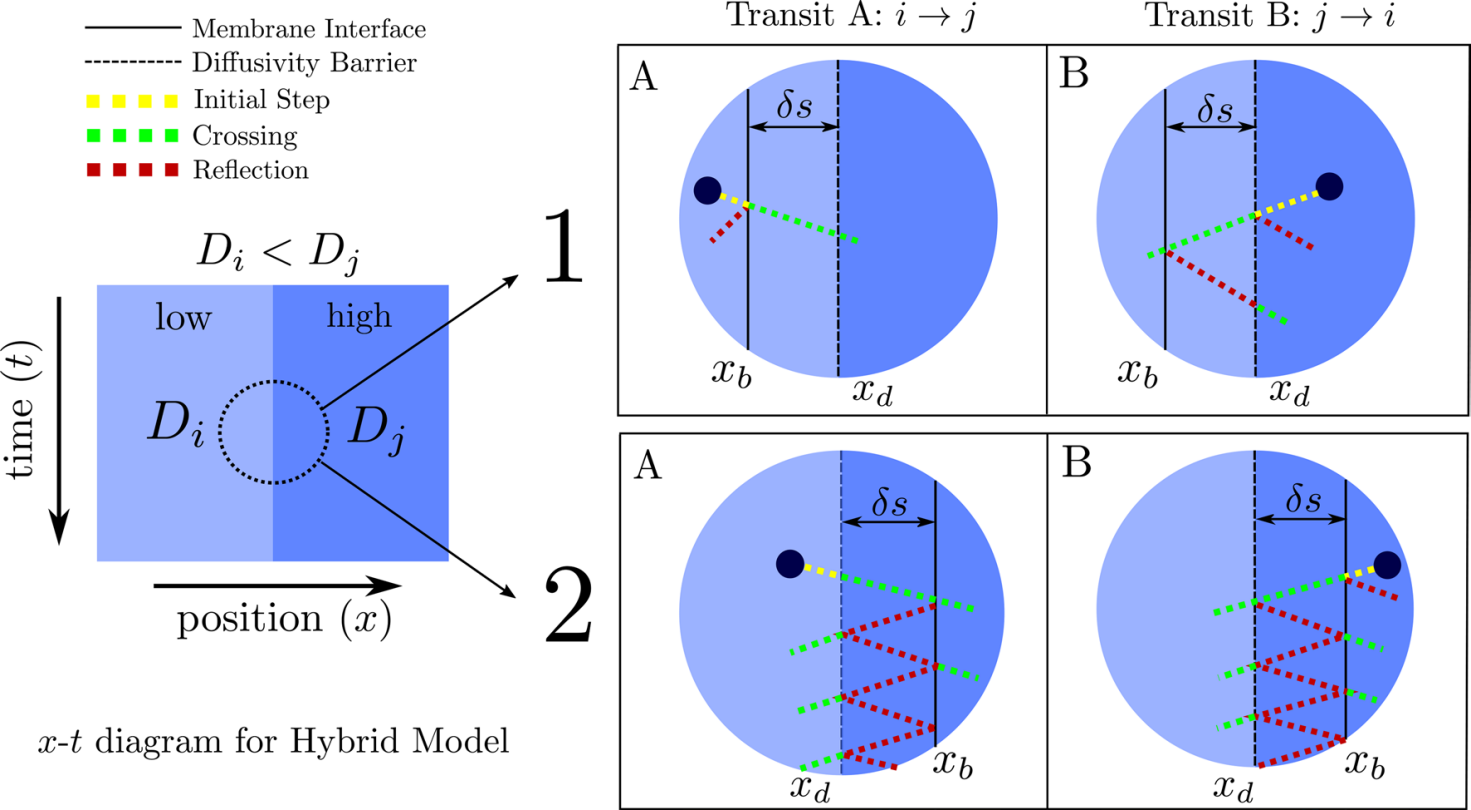
Illustration of the different possible sequences of events when a particle attempts a transit between compartments with low ($D_i$, left) and high ($D_j$, right) diffusivity. Configuration 1 places the membrane in the low diffusivity region ($x_b < x_d$), while configuration 2 places the membrane in the high diffusivity region, ($x_b > x_d$). We illustrate cases A and B, where A refers to an attempted transit from $i \rightarrow j$ and B from $j \rightarrow i$. Note that particles can freely cross the diffusivity interface $x_d$ from low (light blue) to high (dark blue) diffusivity regions, but may be reflected in attempting to cross from high to low diffusivity regions. For case 2, this results in multiple/infinite reflections in the infinitesimal gap $\delta {s}$ between the membrane and the diffusivity jump.

The hybrid model considers an infinitesimal space $\delta {s}$ between the membrane position $x_b$ and the diffusivity change $x_d$. The overall transit probability $p_{t_{\large {hyb}}}$ is derived by calculating the probability of a particle overcoming the two barriers in sequence. As illustrated in Fig. 2, there are two possible configurations depending on the location of the membrane interface $x_b$. The first and second configuration place the interface membrane in the low ($x_b<x_d$) and high diffusivity ($x_b>x_d$) region respectively. We notice that positioning the membrane interface in the high diffusivity region (configuration 2) results in multiple/infinite reflections in the infinitesimal gap $\delta {s}$ between the membrane and the diffusivity jump. In Appendix B we deduce the transit probabilities for both possible configurations and prove that configurations 1 and 2 lead to the same overall transit probability $p_{t_{\large {hyb}}}$. We observe that configuration 1 ($x_b<x_d$) is the preferred arrangement as it simplifies the multiple/infinite reflections observed in configuration 2 and reduces the transit probability $p_{t_{\large {hyb}}}$ to the following simple expression

6 $$\begin{aligned} p_{t_{\large {hyb}}, (i \rightarrow j)} = p_{b, (i \rightarrow j)} \cdot p_{d, (i \rightarrow j)} \quad \text {where} \quad p_{d, (i \rightarrow j)} = \min \left( 1, \sqrt{\frac{D_j}{D_i}} \right) \end{aligned}$$

where $p_{b}$ is the membrane transit probability and $p_{d}$ the probability of a particle when transitioning between two media of different diffusivity. Originally^29^, $p_{d}$ is presented as an elegant interpretation of the behaviour of random walkers when transitioning between media of different diffusivity, whilst $p_{b}$ is given by Eq. (5). Note that, as illustrated in Fig. 2 where the left compartment has a $D_i$ less than the $D_j$ of its right hand neighbour and for the interfaces in configuration 1 ($x_b<x_d$), the probability of an attempted transit from *i* to *j* is simply $p_{t_{\large {hyb}}}=p_{b}$ whereas for an attempted transit in reverse, from *j* to *i*, the probability is $p_{t_{\large {hyb}}}=p_{b} p_{d}$ . In general, for $D_i \le D_j, p_{t_{\large {hyb}}}=p_{b}$ whilst for $D_i > D_j, p_{t_{\large {hyb}}}=p_{b} p_{d}$

### The analytical solution for 1D semi-permeable layered media

In order to compare the performance of both transit models we employ an analytical solution as benchmark. Moutal et al.^9^ presented an elegant transcendental equation $F(\lambda ) :=0$ that is to be solved in order to obtain the eigenvalues $\lambda$ of the diffusion operator. The eigenvalues are the roots of $F(\lambda ^\star )$ where $\lambda ^\star$ is an auxiliary variable considered when *F* is evaluated on a continuous range. The solution assumes the time and space variables in Eq. (1) are separable reducing the eigenvalue problem to the Helmholtz equation.

7 $$\begin{aligned} D_i \, u'' + \lambda u = 0 \quad \forall x \in [b_i, b_{i+1}]. \end{aligned}$$

Using the general solution to this ODE, which is a function of the eigenvalues, one can construct a transcendental equation ensuring that all boundary conditions are satisfied simultaneously. This is achieved through a series of matrix multiplications that link compartments together^9^. The problem of finding eigenvalues is therefore reduced to that of finding the roots of that transcendental equation

8 $$\begin{aligned} F(\lambda ) :=\begin{bmatrix} \kappa _m/\sqrt{\lambda D_{m}} &{} 1\\ \end{bmatrix} {R}_{m}(\lambda ) \left( \prod _{i=1}^{m-1}{ {M}_{i,i+1}(\lambda ) } \right) \begin{bmatrix} 1 \\ \kappa _0/\sqrt{\lambda D_1} \end{bmatrix} = 0 \end{aligned}$$

where ${R}_i(\lambda )$ and ${M}_{i,i+1}(\lambda )$ are specific matrices that depend on the properties of each compartment^9^. For a sensible choice of permeabilities for internal barriers ($0< \kappa _i < \infty$, i.e. no trivial cases), there exist a countably infinite number of real eigenvalues, all of which are non-negative, ordered, and simple^9^:

9 $$\begin{aligned} 0 \le \lambda _{1}< \lambda _{2}< \, \dots \lambda _{N} < \dots \, \lambda _{\infty } \end{aligned}$$

The eigenvalues of higher order have diminishing importance with increasing solution time *t*. For the domains in this work, we use a truncation point of the order of  $\it{N} \le {\mathscr {O}}({10^3})$ as it has been observed to be enough for an accurate and smooth solution. The solution is computed by evaluating and summing the eigenmodes $\nu _n(x)$ throughout the domain at several linearly-spaced points $x_q \in [0, b_m]$

10 $$\begin{aligned} U(x_q, t) \approx \sum _{n=1}^{N}{ e^{-\lambda _n t} \nu _n(x_q) \nu _n(x_{q,0}) } \end{aligned}$$

for an initial condition of a Dirac delta function at $x_{q,0}$.

11 $$\begin{aligned} U(x_q, 0) = \delta (x_q - x_{q,0}) \end{aligned}$$

As described above, the eigenvalues (roots of Eq. (8)) are found numerically up to to $\lambda _{N}$ where $\it{N}=1000$. The solution $U(x_q, t)$ is, therefore, semi-analytical. As a consequence, numerical errors introduced by the root finding procedure manifest themselves as errors in the solution. Due to the linearity of the diffusion equation, a uniform initial condition in a certain region can be solved by adding and normalising all the solutions obtained by several delta Dirac functions within the interval of interest.

### The diffusion problem applied to DWI in the heart

Although the diffusion problem is general, we illustrate the performance of both models using biological parameters pertaining to cardiomyocytes. In order to investigate the applicability of our new hybrid model mentioned in “Hybrid model”, we investigate the transient and steady state behaviour of $U(x, t; x_0)$ in a two compartment domain. We compare the diffusion flux and numerical results with a semi-analytical solution and a reference transit model^25,26^. A more complex example is provided comparing the performing of both models in the measurement of diffusion in the heart using diffusion weighted imaging (DWI).

#### Transient and steady state analysis of diffusion flux in a two compartment domain

We analyse the flux of particles crossing the membranes that is represented by the flux boundary condition in Eq. (3). This equation relates the flux *J* to the permeability and the difference in concentrations across the barrier. The units of *J* are concentration (fraction of walkers) per unit time and area, but we omit the latter such that $[J] = 1/\hbox {ms}$. This flux boundary condition, allows us to compute the instantaneous analytical flux evaluating U on either side of the discontinuity and the instantaneous numerical flux by measuring the fraction of particles that cross the barrier for each specific time step. The net instantaneous flux is obtained each time step by subtracting the fluxes from either side (left/right) and the cumulative flux (flux integral) throughout the simulation. Based on the analytical and numerical flux comparison, we perform two different types of analysis to evaluate the efficacy of the transit models. We implement a steady and transient state analysis by considering a total and partial uniform distribution. We determine the relative error $\epsilon _{global}$ for both transit models using the cumulative flux  $\mathscr {J}(t) = \int _{0}^{t}{J(\tau ) \hbox {d}{\tau }}$ which represents the net concentration of walkers that has crossed the membrane up until *t* and approaches 0.5 as $t \rightarrow \infty$ to match the steady state solution. This global relative error $\epsilon _{global}$ measures the area in between the analytical and numerical solution during the entire simulation. We utilise this global relative error to investigate the time step dependence and compare the performances of our new hybrid model and the reference model^25^ under the influence of different permeability and diffusivity values.

#### The diffusion problem applied to a synthesised histology-based domain

The domain has been obtained from sections of swine myocardium, cut perpendicular to the long axis of the cardiomyocytes. We have used automatic segmentation developed in previous work^30^ to obtain a distribution of cell sizes. We have utilised this previous work to find statistics parameters (mean cross-sectional area, standard deviation) to recreate a synthesised histology-based domain assuming a circular cross-sections for cardiomyocytes^31^. The cell areas are converted to diameters which we use as intra-cellular compartment lengths in the 1D domain. The extracellular space is recreated considering a uniform distribution.

Few estimates exist in literature for the free diffusivity in the myocardium and/or the permeability of the cardiomyocyte membrane. A recent study^32^ observed diffusivity values of ${1.2}\,\upmu \hbox {m}^2/\hbox {ms}$ and ${3}\,\upmu \hbox {m}^2/\hbox {ms}$ in the intra- and extra-cellular space of heart rat cells. In general it should be expected that the compartment-specific bulk diffusivity are lower than the free diffusivity of water and $D_\text {ICS}<D_\text {ECS}$ due to the concentration of sub-cellular structures. For the numerical simulations, we have considered $D_\text {ICS} = {0.5}\,\upmu \hbox {m}^2/\hbox {ms}$,  $D_\text {ECS} = {2}\,\upmu \hbox {m}^2/\hbox {ms}$ and $\kappa = {0.05}\,\upmu \hbox {m}/\hbox {ms}$. The cell membrane permeability has been estimated from measurements of apparent exchange rate ($1/\tau _\text {ex}$). Exchange rates are commonly reported in a range of ${6}\,\hbox {to}\,{30}\,\hbox {Hz}$^32,33^, but have been found as high as ${50}\,\hbox {Hz}$ for healthy leg muscles of rats^34^.

We perform the random walk simulations with $N_p = {10^6}$ walkers and a time step of $\delta {t} = {1.5}\hbox {ms}$. This time step intentionally exceeds $\delta {t}_{\text {max ref}}$ required for an accurate handling of transit using the reference model in Eq. (5), while still limiting walkers to a single barrier interaction per step $\delta {t}={1.5}\,\hbox {ms}<\delta {t}_{\text {max domain}}$. Random walk simulations are run for $t = {1000}\,\hbox {ms}$ and the analytical solution is evaluated for the same space and time parameters. We use the semi-analytical solution described in “The analytical solution for 1D semi-permeable layered media” to obtain 1000 eigenvalues with $\lambda ^\star \in [0, 500]$. Conversely to “Flux diffusion analysis: transient state”, the transient solution $U(x, t; x_0)$ is obtained by seeding all the walkers at the centre of the domain, while the steady-state solution is initialised by seeding the walkers uniformly.

#### Measurement of diffusion: diffusion weighted imaging (DWI) signal

There are many comprehensive reviews of DWI and the measurement of diffusion using MRI^35^. Briefly, Diffusion Weighted Imaging (DWI) is a method of contrast generation based on the self-diffusion (Brownian motion) of water molecules (hydrogen spins) present in the biological tissue. In DWI, the magnetisation of the hydrogen spins align with the magnetic field $B_0$ and precess at a frequency $w=-\gamma B_0$ according to their gyromagnetic ratio  $\gamma = {267.5}\times 10^{6}\,\hbox {rad}/(\hbox {s}\,\hbox {T})$. Radio-frequency pulses are used to rotate the spin magnetisation away from their initial direction and spatial gradients in the magnetic fields *G*(*t*) modify the precessional frequency and therefore phase according to Eq. (12).

12 $$\begin{aligned} \phi _p(t) = \gamma \int _{0}^{t}{ G(\tau ) x_p(\tau ) \hbox {d}{\tau } } \end{aligned}$$

In DWI the imaged sample is subjected to a sequence of two symmetric and effectively opposite polarity gradients of strength $G_{max}$ and duration $\delta$ separated by a diffusion time $\Delta$ to encode the distances diffused into the received signal. The accumulated precessional phase of the magnetisation vector of each spin at the end of the DWI sequence is directly related to the distance that each spin has diffused during $\Delta$. The signal *S* obtained after diffusion encoding is determined by the sum of the incoherent spin magnetisation vectors. The signal attenuation is the ratio between the signal recovery *S* and the initial signal $S_0$ that we would obtain if the spins were static. By means of the narrow pulse approximation (NPA)^35,36^ it is possible to estimate the DWI signal directly. This assumes that the gradients are applied instantaneously, i.e. $\delta \rightarrow 0$ as the wave number $q(t) = \gamma \int _{0}^{t}{ G(\tau ) \hbox {d}{\tau } }$ remains finite ($=\gamma G \delta$). In DWI, the strength and timing of the gradients is described by the b-value. In the case of NPA, the b-value is calculated as $b=q^2 \Delta$. Through the Bloch–Torrey equations^37^, it is possible to prove an exponential relationship ($S/S_0=\exp ({-bADC})$) between the signal attenuation and the apparent diffusion coefficient (*ADC*) (which is affected by both the compartmental diffusivities, barrier density and permeability).

The analytical and random walk methods presented previously allow us to solve for the diffusion propagator. For the random walk simulations, we seed the walkers uniformly in the histology-based domain and we let them diffuse for a period of 1 s. The numerical simulations compute the accumulated precessional phase of each walker/spin at the end of the sequence and the narrow pulse approximation allows the precessional phase to be calculated based on the displacement during the diffusion time. The signal attenuation can then be directly computed using the following equations

13 $$\begin{aligned} S_{\rm{rw}}(\Delta , q)= & {} \frac{1}{N_p} \left| \sum _{p}^{N_p}{e^{-\imath q \left( x_p(\Delta )-x_p(0) \right) } } \right| \end{aligned}$$

14 $$\begin{aligned} S_{\rm{ana}}(\Delta , q)= & {} \frac{1}{\sum _{i=1}^{m}{L_i}} \sum _{n}{ e^{-\lambda _n \Delta } \left| {\int _{\Omega }{ \nu _n(x) e^{\imath q x} \hbox {d}{x} } }\right|}^2 \end{aligned}$$

where $\imath$ denotes the imaginary unit $\sqrt{-1}$. The analytical attenuation signal is calculated using an expression derived for uniform initial seeding^9^. We approximate the integrals numerically using trapezoidal integration over a finely-discretised domain. We analyse and compare the signal attenuation and apparent diffusion coefficient (ADC) with analytical results for a large time step of $\delta {t}= {1.5}\,\hbox {ms}$ and a small time step $\delta {t} = {0.01}\,\hbox {ms}$. In order to provide insights in other potential applications, we consider a wide range of permeabilities (0 to ${1}\,\upmu \hbox {m}/\hbox {ms}$) apart from the biological ambit. We have considered a b-value of ${1}\,\hbox {ms}/\upmu \hbox {m}^2$ for all the simulations.

## Results

### Convergence

In this work histograms are used to illustrate the random walk solution, density-normalised such that $\rho _\text {bin} = c_\text {bin}/N_p/w_\text {bin}$ where $c_\text {bin}$ and $w_\text {bin}$ are the bin count and width respectively. In Appendix D, we study the convergence of the random walk simulations for a simple two-compartment domain with varying numbers of walkers and time steps^38^. We set $D_L = {0.5}\,\upmu \hbox {m}^2/\hbox {ms}$, $D_R = {2.5}\,\upmu \hbox {m}^2/\hbox {ms}$, and $\kappa = {0.05}\,\upmu \hbox {m}/\hbox {ms}$ as these are feasible parameters in cardiac tissue and comparable to the values used for the remaining analyses. We conclude that $N_p = {10^6}$ walkers is sufficient to obtain an accurate solution.

### Analysis of the steady state

**Figure 3:**
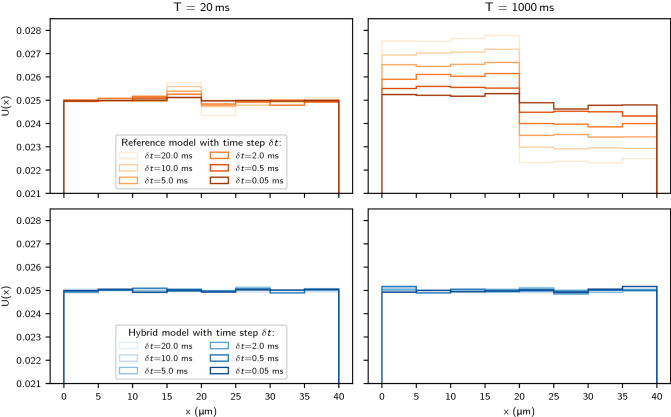
Histograms of walker positions after random walk simulations of the steady state. Initial positions were sampled from a uniform distribution to seed walkers with a constant density throughout the domain. We have performed the simulations with $N_p = 10^6$. We applied the reference membrane model (top) described in Eq. (5), with permeability $\kappa = {0.05}\,\upmu \hbox {m}/\hbox {ms}$, and the new hybrid model (bottom) from Eq. (6). Simulations were performed with varying step sizes $\delta {t}$ for a short and a long simulation time *T*. We consider $D_L = {0.5}\,\upmu \hbox {m}^2/\hbox {ms}$, $D_R = {2.5}\,\upmu \hbox {m}^2/\hbox {ms}$ and $L = {20}\,\upmu \hbox {m}$.

We investigate the time step dependence of the membrane transit model for a two-compartment domain. Figure 3 shows random walk solutions using the new hybrid model and the reference transit model^25^. We examine different time steps $\delta{t}$ (${20,10,5,2,0.5, \text{and }} {0.05}\,\hbox {ms}$) and two solution times ${20\, \text {and } 1000}\,\hbox {ms}$. In the steady state $U(x, t) = \text {const}$. As a result the expected particle density for each bin irrespective of time step $\delta {t}$ should be $\rho _{\text {expected}} = 1/\sum {L} = 0.025$. For the short time solution $T = {20}\,\hbox {ms}$, the reference transit model fails to preserve the initial steady-state solution near the internal barrier at $x = {20}\,\hbox {ms}$. As the time increases, a concentration imbalance develops across the interface.

We observe that the difference in *compartment* density $\Delta {U}$ across the membrane increases with $\delta {t}$ and appears to stabilise at some value as $t \rightarrow \infty$: For $\delta {t} = {0.05}\,\hbox {ms}$, the reference model shows an excess of concentration in the left compartment of ${0.88}\%$, for $\delta {t} = {20}\,\hbox {ms}$ this increases to ${10.2}\%$. In Appendix C, we mathematically prove the limitations of the reference model. On the other hand, the new hybrid model produces the expected solution with a constant *U* with a maximum of $\Delta {U} = {0.18}\%$ for all values of *t* and $\delta {t}$, thus suggesting that there is no accumulation of walkers for any time step. This variation in the density between bins for the hybrid model is attributed to randomness of the simulation as the maximum deviation is $\pm {0.437}\%$ relative to $\rho _{\text {expected}}$ considering a ${95}\%$ confidence interval.

### Flux diffusion analysis: transient state

We perform two different transient-state analyses to independently investigate the influence of the step size and the influence of several values for the permeability and diffusion coefficients.

#### The influence of step size

**Figure 4:**
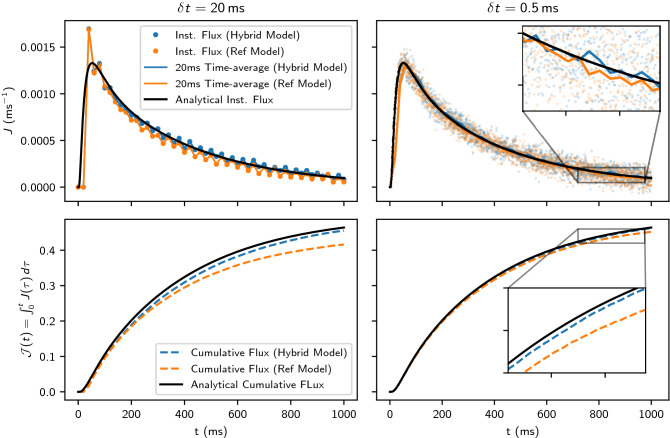
Top figures: instantaneous (numerical/analytical) and time-averaged (plotted as the average over intervals of $\Delta {t} = {20}\,\hbox {ms}$) fluxes *J*(*t*) through the membrane as a function of simulation time. Bottom figure: Analytical and numerical net cumulative flux. We show results for two different step sizes $\delta {t}$: 20 and 0.5$\,\hbox {ms}$. Domain and simulation parameters are $D_L = {0.5}\,\upmu \hbox {m}^2/\hbox {ms}$, $D_R = {2.5}\,\upmu \hbox {m}^2/\hbox {ms}$, $\kappa = {0.05}\,\upmu \hbox {m}/\hbox {ms}$, $L = {20}\,\upmu \hbox {m}$, $N_p = 10^6$.

Considering the same parameters introduced in the previous section “Analysis of the steady state”, we perform simulations up to $t = {1000}\,\hbox {ms}$ using the largest and a small time step $\delta {t}$: ${20 \text { and } 0.5}\,\hbox {ms}$. Figure 4 shows the instantaneous and cumulative fluxes obtained from the random walk simulation at every time step alongside the analytical solution. For the random walk solution, we also plot a time-averaged flux over fixed intervals of $\Delta {t} = {20}\,\hbox {ms}$ to allow for comparison between both plots. Note that the time-average flux and instantaneous flux coincide for the largest time step as $\delta {t} = \Delta {t} = {20}\,\hbox {ms}$.

The (analytical) flux *J* rapidly increases early in the simulation and peaks at $t = {58.53}\,\hbox {ms}$. As *t* increases, walkers continue to cross the membrane towards the initially empty compartment ($J > 0$ always). We observe that for a large time step $\delta {t} = {20}\,\hbox {ms}$, the time-averaged/instantaneous flux for both transit models over-estimate the initial peak in magnitude. However, if we compare the cumulative fluxes at the final end-point of the simulation ($t = {1000}\,\hbox {ms}$), the reference model underestimates the tail with a relative error of ${-10.27}\%$ compared to an error of ${-1.87}\%$ for the hybrid model. We observe that reducing the time step increases the number of walkers crossing the membrane increasing the overall convergence. For $\delta {t} = {0.5}\,\hbox {ms}$, the relative error at the end of the simulation is reduced to ${-2.6}\%$ and to ${-0.46}\%$ for the reference and hybrid model respectively. This is consistent with the results observed in Fig. 3 for the steady-state analysis.

#### The influence of permeability and diffusivity

In order to study the effects of permeability and diffusivity, we consider the simple domain varying the ratio ($D_R/D_L$) between diffusivities in either side. We consider a constant left diffusivity of $D_L = {2}\,\upmu \hbox {m}^2/\hbox {ms}$ and a range of ratios ($0.05< D_R/D_L < 2.5$). We consider two permeability values (0.05 and 0.5$\,\upmu \hbox {m}/\hbox {ms}$) that correspond to exchange times of the order of ${50}\,\hbox {Hz}$ and ${500}\,\hbox {Hz}$ respectively. The first permeability (${0.05}\,\upmu \hbox {m}/\hbox {ms}$) is linked to exchange times that are closer to what we would observe in human cells^32^ and the second permeability value covers higher exchange rates that might be useful for other applications. We perform simulations up to $t = {1000}\,\hbox {ms}$ for seven different time steps $\delta {t}$: 40,8,4,2,0.5,0.1, and 0.05$\,\hbox {ms}$ and nine different varying diffusivity ratios 2.5, 1.8, 1.6, 1, 0.4, 0.2, 0.1, and 0.05. Note that the largest $\delta {t} = {40}\,\hbox {ms}$ considered for the simulations coincides with the most restrictive $\delta {t}_{\text {max domain}}$ given by $D_R/D_L = 0.05$ amongst all diffusivity ratios.

**Figure 5:**
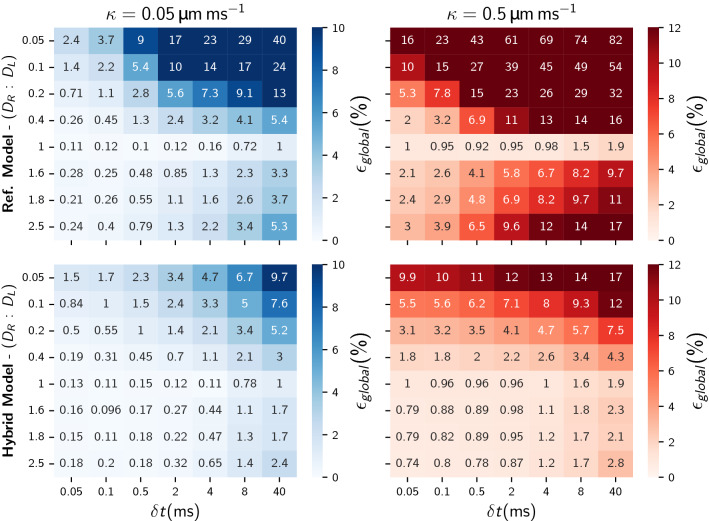
Global errors using the reference (upper row) and hybrid transit model (lower row) for different time steps $\delta {t}$, distinct diffusivity ratios $D_L:D_R$ and two different permeabilities $\kappa$. The global error measures the accuracy of the numerical simulation by calculating the area between the analytical and numerical cumulative flux during the entire simulation. Simulations were run until $t = {1000}\,\hbox {ms}$ with $N_p = 10^6$ walkers seeding the walkers in a partial uniform region in the left compartment $x_0 \in [6, {14}\,\upmu \hbox {m}]$.

Figure 5 shows the global relative errors that have been computed by evaluating the integral difference between the numerical and analytical cumulative flux. The top plots and the bottom plots illustrate the global errors for the reference model and hybrid model respectively. As mentioned in “Transient and steady state analysis of diffusion flux in a two compartment domain”, each simulation has been performed initialising the walkers in a partial uniform region within the left compartment. For a constant permeability, we observe that the global errors escalate as we increase the difference between diffusivities. These increments are consistently lower for the hybrid model as it includes the probability when transitioning between two different media through Eq. (6). Similarly to Fig. 4, lowering the step size increases the number of walker collisions with the membrane resulting in an overall faster convergence and accuracy. As it can be observed in Eq. (6), the hybrid model incorporates the reference model to solve the membrane/interface probability. In Appendix C, we show that the reference model does not preserve the interface reflection and leads to a permeability-related error. As a result, both models show a permeability error dependency, however, the hybrid model relative errors are consistently lower due to the initial error reduction in the diffusion media. The dependencies of the accuracy ($\epsilon _{\text {global}}$) on the time step ($\delta {t}$) shows that the improvement in simulation efficiency provided by the hybrid model is greatest for highly permeable membranes and large diffusivity gradients. As an example, for $\kappa ={0.5}\,\upmu \hbox {m}/\hbox {ms}$ and (i) $D_R:D_L=0.05$, (ii) $D_R:D_L=2.5$ similar accuracies (i) $\epsilon _{\text {global}} \approx {16}\%$ and (ii) $\epsilon _{\text {global}} \approx {2.8}\%$ are obtained with $\delta {t} = {40}\,\hbox {ms}$ and $\delta {t} = {0.05}\,\hbox {ms}$ using the reference and hybrid model respectively. As a consequence, for a similar accuracy the hybrid model reduces the simulation time by a factor of 190 from $\approx {950}\,\hbox {s}$ to $\approx {5}\,\hbox {s}$. For the low permeability case $\kappa ={0.05}\,\upmu \hbox {m}/\hbox {ms}$ and highest diffusivity ratio $D_R:D_L=0.05$ accuracies of $\approx {3.5}\%$ are obtained with $\delta {t}={2}\,\hbox {ms}$ using the hybrid model while the reference model requires a lower time step $\delta {t}={0.1}\,\hbox {ms}$. In this case the computational time is a factor of 5 lower, which although reduced from 190, is still substantial.

### Transient and steady-state diffusion solutions in the histology-based domain

**Figure 6:**
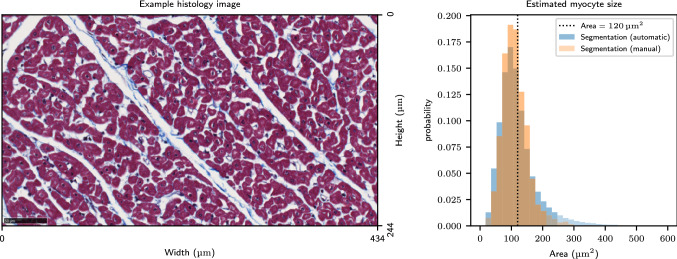
Illustration of the process of synthesising a 1D domain from histology data. Left figure: An example of a region of histology from a wide-field microscopy image. This is part of a large stack of histology slices obtained from the mesocardium of swine. Cardiomyocytes (red–purple) are cut perpendicular to their long axis. Extra-cellular space is white, while collagen is stained blue. Right figure: Distribution of cell sizes from automatic segmentation for the entire stack of images as well as manual labelling of a small representative region.

**Figure 7:**
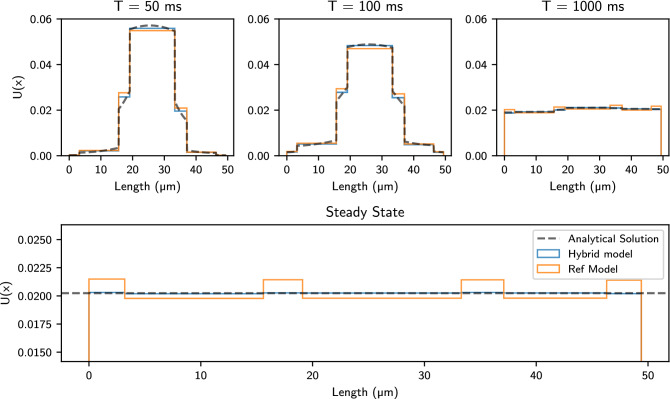
Analytical and random walk solutions using $N_p = 10^6$ walkers and a time step of $\delta {t} = {1.5}\hbox {ms}$. Top: Transient solution $U(x, t; x_0)$ for initial concentration at $x_0$ located in the centre of the domain considering three simulation times ${50,100\,\text{and}\,1000}\,\hbox {ms}$. Bottom: Steady-state solution obtained after uniformly seeding the walkers in the domain. The simulation time is set to $T = {1000}\hbox {ms}$. The reference model results in accumulation of walkers in ECS compartments. Note that this effect is visually amplified by the choice of axis data range.

Figure 6 illustrates the distribution of cell sizes for the manual and automatic segmentation^30^. As explained in  ”The diffusion problem applied to a synthesised histology-based domain”, we recreate a histology-based domain by considering circular cross-sections for cardiomyocytes. The segmentation data shows a mean cross-sectional area of $\mu = {120}\,\upmu \hbox {m}^{2}$ and a standard deviation of $\sigma = {40}\,\upmu \hbox {m}^{2}$. We utilise these parameters to create a synthesised histology-based domain using a normal distribution ($\mu \pm 2 \sigma$) for the intra-cellular space and a uniform distribution (3–${5}\,\upmu \hbox {m}$) for the extra-cellular compartments. The resulting domain is recreated by drawing both intra-cellular and extra-cellular distributions until reaching a total length of ${49.5}\,\upmu \hbox {m}$. Figure 7 shows the final steady state and three different transient states for the histology-based domain. The transient states are analysed at three different diffusion times $T = {50,100,\,\hbox {and}\,1000}\,\hbox {ms}$ considering that all the particles are initialised in the middle of the domain $x_{0} = {24.75}\,\upmu \hbox {m}$. We observe good agreement between the hybrid model and the analytical solutions. We notice that the reference model tends to overestimate the flux through the barriers in the initial transient states leading to higher concentrations in the ECS. This accumulation of walkers is present and is carried during the entire simulation until reaching the steady-state. This excess of walkers in the ECS persists throughout the simulation and is consistent with the findings in “Analysis of the steady state” and “Flux diffusion analysis: transient state”.

### DWI results

**Figure 8:**
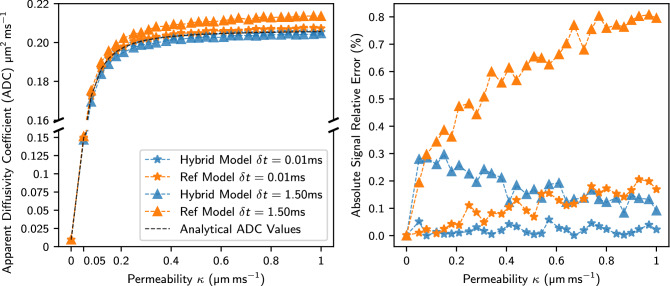
Absolute relative signal error and ADC values for a wide range of permeability values considering a very small ($\delta {t} = {0.01}\,\hbox {ms}$) and large ($\delta {t} = {1.5}\,\hbox {ms}$) time step. All the simulations consider $N_p = 10^6$ walkers, $D_\text {ECS} = {2}\,\upmu \hbox {m}^2/\hbox {ms}$ and $D_\text {ICS} = {0.5}\,\upmu \hbox {m}^2/\hbox {ms}$.

We analyse how the diffusion errors observed in  “Transient and steady-state diffusion solutions in the histology-based domain” affect the DWI signal and apparent diffusion coefficient (ADC). We compare the signal attenuation and ADC with the theoretical analytical results. As mentioned in “Measurement of diffusion: diffusion weighted imaging (DWI) signal”, we seed the walkers uniformly and consider the narrow pulse approximation (NPA). Figure 8 shows the ADC and the absolute signal relative error for a large interval of membrane permeabilities and two different step sizes. Equation (13) shows the relation between the numerical signal error and the displacement of the walkers. Similar to the flux analysis, we notice a strong dependency between the permeability and the accuracy of both transit models. If we compare the errors for increasing permeability values, we observe that the reference model progressively becomes less accurate. As illustrated in Fig. 8, we observe low relative errors ($\approx {0.3}\%$) for $\delta {t} = {1.5}\hbox {ms}$ that scale up to ${0.8}\%$ for the reference model. These signal and ADC errors can be reduced when decreasing $\delta {t}$ due to the greater number of particle collisions with the membranes. In the application that we are interested in, the cardiomyocytes have low permeability values ($\kappa < {0.05}\,\upmu \hbox {m}/\hbox {ms}$)^34^ where both transit models perform similarly with low relative errors.

## Discussion

Simulating the transit of particles through a semi-permeable membrane separating media with differing diffusivities can be achieved using analytical or numerical techniques. In this work, we present novel methods for determining the particle density in a 1D domain and the flux through a membrane.

Monte Carlo random walk based method are frequently used to solve the diffusion equation (1) in a range of applications. When a walker encounters a barrier such as a semi-permeable membrane, the probability of transit needs to be accurately computed and the walker step size must be adjusted when transiting between compartments of differing diffusivities^11^. One of these so-called transit models is the popular method described in Eq. (5)^25^. As described in the original paper, this reference transit model^25^ requires a highly resolved time step. For this reason, it is suggested that the maximum time step ($\delta {t}_{\text {max ref}}$) is limited by a maximum possible transmission probability of 0.01^25^. This can be a computational challenge for long and even short diffusion time simulations when other Monte Carlo parameters such as the number of walkers $N_p$ or the number of unique experiments need to be considered. In this work we propose a new hybrid transit model with the motivation of lifting this restriction. The new model that we present is based on treating the membrane as an infinitesimal space such that the diffusivity gradient and the membrane permeability can be considered as two independent factors. It is important to note that the hybrid model is built on top of the reference model as it is used to solve the membrane probability. Using an analytical solution as a gold standard, we have studied the accuracy of the hybrid and reference transit model^25^ when exceeding the maximum step size ($\delta {t}_{max}$). We have assessed both models by analysing the membrane flux varying several parameters in a simple domain.

We have found that the reference model leads to errors in the net migration of walkers and results in concentration imbalances for steady-state solutions. Further analysis in Appendix C.3 demonstrated that the transit model inherently tends to accumulate walkers when the membrane divides two compartments of different diffusivities. In Appendix C we also note that the interface reflection condition^11^ is not respected as $\kappa \rightarrow \infty$ and this is consistent with the findings observed for both transit models in Fig. 5 and with the relation between diffusivity/permeability to fit within the maximum time step ($\delta {t}_{max}$).

The numerical simulations performed in this study consider a fixed step size. In “Flux diffusion analysis: transient state”, we have considered a partial uniform distribution of walkers in the left compartment to avoid sampling error for large step sizes due to the unique number of possible walker positions. From the numerical simulations in the simple domain, we numerically prove the limitations of the reference model for varying diffusivities. From our steady-state and transient findings, we conclude that these imbalances increase for higher step sizes and longer diffusion times. Further numerical analysis shows that the hybrid model substantially reduces the diffusivity and permeability-related errors present in the reference model and imply that our model is computationally more efficient as similar accuracies using the reference model are linked to lower step sizes $\delta {t}$ and larger computational runtimes. Simulations show the computational time depends on both permeability and diffusivity. Moreover simulations reveal that the hybrid model offers substantial gains in computational efficiency, reducing run times by a factor of 5 for simulations in tissue to a factor of $\sim$ 200 in other applications. It is important to note that when there is no diffusivity discontinuity the hybrid model is identical to the reference model.

One important application of the methods presented in this work is in the simulation of diffusion weighted imaging (DWI). Previous numerical simulations of diffusion tensor imaging (a variant of DWI) in the heart were based on impermeable membranes^22^ and resulted in more anisotropic diffusion than commonly found in imaging studies^39^. Initial work using finite volume methods^40^ has suggested that this overestimation of anisotropy is likely due, at least in part, to the failure to include permeability within the model.

The simulations in “Transient and steady-state diffusion solutions in the histology-based domain” use a domain constructed based on microscopy images of histology sections. Using a previously developed method^30^ to automatically segment cells, we have extracted characteristic cell sizes to generate a representative one-dimensional domain. A mean cell area of ${120}\,\upmu \hbox {m}$, which corresponds to a diameter of ${12.4}\,\upmu \hbox {m}$ assuming circular cells was obtained from segmentation of the data. This value is slightly lower than the reported range of 17 to ${25}\,\upmu \hbox {m}$ in^31^. However, tissue is known to shrink during histological preparation and this may be compensated for during image processing by morphing the domain^22^.

The cardiomyocyte membrane permeability ($\kappa ={0.05}\,\upmu \hbox {m}/\hbox {ms}$) and the diffusivity values ($D_\text {ECS}={2}\,\upmu \hbox {m}^2/\hbox {ms}$, $D_\text {ICS}={0.5}\,\upmu \hbox {m}^2/\hbox {ms}$) in the histology-based simulations limit the $\delta {t}_{\text {max ref}}$ to extremely low values $\delta {t} \approx {0.002}\,\hbox {ms}$. Similarly to the simple domain analysis, our histology-based results for the reference model show accumulation of particles in the ECV and these errors are carried into the DWI signal computation. We have observed that both models have very small and similar errors for low permeability in the range of cardiomyocytes. We have shown increasing errors in the results of the reference model with larger permeability values. The findings regarding the limitations of the reference model at high $\kappa$ and larger ratios of *D* between compartments are particularly important for applications beyond biological tissue such as heat transfer where different sets of parameters may be required. For example, a thin layer of material in heat conduction problems can be modelled as a membrane with permeability *D*/*L*.

In this work, we have reduced the complexity of the simulations to a one dimensional histology-based domain. Analytical techniques have been used to compare the performance of our new transit model with a previously random walk transit model. We have extended and validated these findings by performing 2D simulations on a representative domain of the cardiac microstructure. An accurate finite volume solution has been used as a gold-standard to assess the performance of our new transit model. We have observed low relative error values extending the application of our transit model to higher dimensions.

## Conclusions

Modelling diffusion within non-simple layered media requires a correct treatment of the particle transit at membranes when numerical solutions are employed.

We investigate the accuracy of a previously proposed transit model for varying diffusion coefficients in the presence of permeable barriers. We compared the numerical results to an analytical method and show the limitation of this reference transit model. We propose an alternative to this problem by considering a hybrid transit model that treats the transitioning between different media and the permeable membrane as two separate probabilities. By comparing numerical and analytical simulations, we conclude that the new transit model performs with lower errors and successfully lifts the step restriction saving overall simulation time and reducing the accumulation of walkers observed in the reference model. While random walk methods for the solution of the diffusion equation in layered media have a number of potential applications, we have considered simulating diffusion-weighted imaging in cardiac tissue.

For the given range of extracellular compartment lengths we find that the time step is already very restricted by the domain itself as the walkers cannot cross multiple compartments. We conclude that in application to cardiac tissue, both transit models present very small and similar errors in the apparent diffusion coefficient, an important integral quantity for diffusion-weighted imaging. However, in other applications where the compartments are larger and both permeabilities and diffusivites are higher, the potential of this new hybrid model can be fully realised. In these cases, the hybrid model becomes a computationally more efficient and more accurate alternative for solving the diffusion equation than existing models.

## Supplementary Information

Supplementary Information.

## Data Availability

The datasets used and/or analysed during the current study available from the corresponding author on reasonable request.
