# Supplementary material for: Random walk diffusion simulations in semi-permeable layered media with varying diffusivity
Source: Sci Rep. 2022 Jun 24;12:10759. doi: 10.1038/s41598-022-14541-y (PMC9232609; doi:10.1038/s41598-022-14541-y)
Supplement: Supplementary file 1 — Supplementary Information. [file 41598_2022_14541_MOESM1_ESM.pdf]

A Reference transit model derivation

The work in[10] presents a derivation of the transit probability p_t for walker positions on a lattice based on a constant step length in the entire domain. Here, we consider domains where the diffusivity between compartments may differ and re-derive the probability of transit for the domain in figure 9 considering the different step lengths.

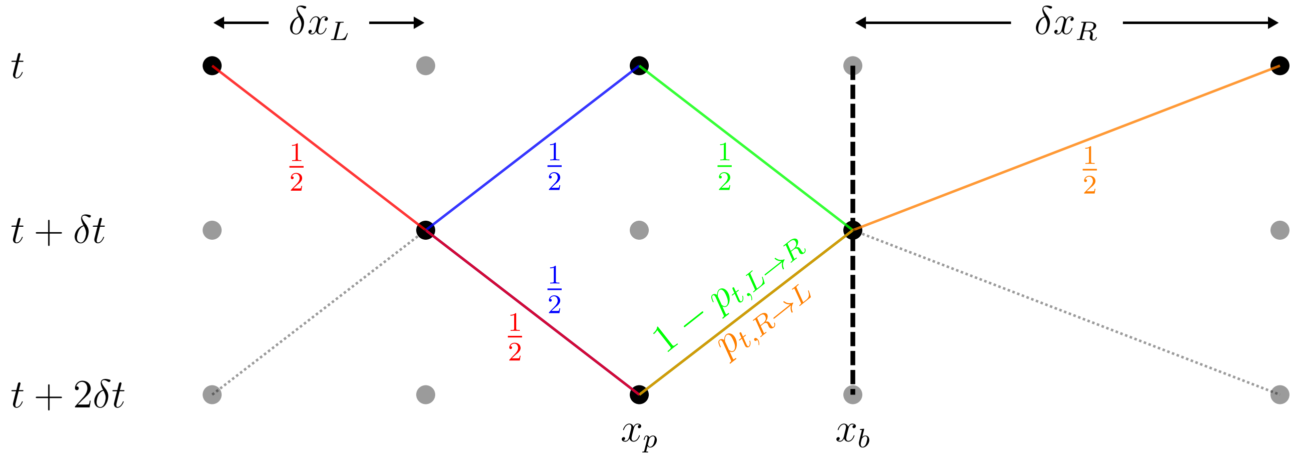

Figure 9. A grid of walker positions x near a permeable barrier located at x_b. The concentration at x_p, i.e. $U(x_p,t+2\delta t)$, is composed of the contribution of three different walker positions at time t (two time steps prior) through four different paths. The green path reflects at the barrier with probability $1-p_{t,L\to R}$, while the orange path passes through the barrier with transit probability $p_{t,R\to L}$.

Consider the probability of finding a walker at x_p as the sum of probabilities of neighbouring walkers jumping to x_p. Away from interfaces, this results in

$$U(x_p,t+\delta t) = \frac{1}{2}U(x_p-\delta x,t)+\frac{1}{2}U(x_p+\delta x,t)\,. \tag{15}$$

Selecting x_p to be near a barrier at x_b (see figure 9 for the possible paths of walkers to reach this position) results in additional terms involving p_t (both $p_{t,L\to R}$ and $p_{t,R\to L}$). The barrier location is chosen to coincide with a grid point. A walker located here will proceed left/right according to the appropriate probability of transit. The contributions of different $U(x,t)$ terms to $U(x_p,t+2\delta t)$ in figure 9 (with the path colour indicated) are:

$$U(x_p,t+2\delta t) = \underbrace{\frac{1}{4}U(x_p-2\delta x_L,t)+}_{\text{free diffusion (red)}}\ \underbrace{\frac{1}{4}U(x_p,t)}_{\text{free diffusion (blue)}}\ +\underbrace{\frac{1-p_{t,L\to R}}{2}U(x_p,t)}_{\text{reflection (green)}}+\underbrace{\frac{p_{t,R\to L}}{2}U(x_p+\delta x_L+\delta x_R,t)}_{\text{transit (orange)}} \tag{16}$$

One may now express x_p relative to x_b, e.g. the contribution of the orange path from the right side of the barrier is:

$$U(x_p+\delta x_L+\delta x_R,t) = U(x_b+\delta x_R,t) \tag{17}$$

This allows us to perform a Taylor series expansion of every term around x_b, taking care to expand infinitesimally to the left and right of the barrier as appropriate.

Following that, we apply the diffusion equation (1) to remove the time derivative. The boundary condition in equation (3)

$$D_R \left.\frac{\partial^2 U}{\partial x^2}\right|_R = D_L \left.\frac{\partial^2 U}{\partial x^2}\right|_L \tag{18}$$

allows us to express everything in terms of $U|_L$, $(\partial U/\partial x)_L$, and $(\partial^2 U/\partial x^2)_L$ as well as $p_{t,L\to R}$ and δx_L. Since we did not specify any constraints regarding the values of D_L and D_R or their relationship, p_t is equivalent to $p_{t,i\to j}$ in equation (5) given the diffusivities $D_L = D_i$ and $D_R = D_j$.

The final expression is:

$$0 = 2p_t\left(1-\sqrt{\frac{D_L}{D_R}}\right)U|_L + 2\left(\left(1-\left(1+\frac{D_L}{D_R}\right)p_t\right)\delta x_L - p_t\sqrt{\frac{D_L}{D_R}}\frac{D_L}{\kappa}\right)\left.\frac{\partial U}{\partial x}\right|_L + p_t\left(1-\sqrt{\frac{D_L}{D_R}}\right)\delta x_L^2\left.\frac{\partial^2 U}{\partial x^2}\right|_L \tag{19}$$

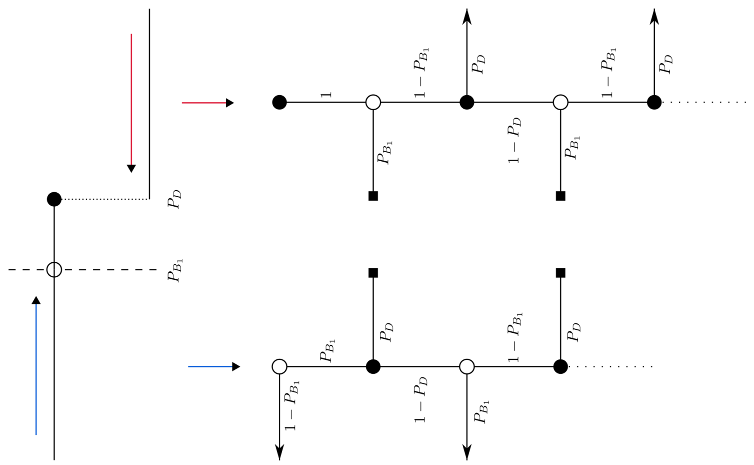

Figure 10. Interface model for a membrane on the fast side

It is important to recognise that this recovers the expression derived by Powles et al. in the case $D_L = D_R$[10]. It also suggests that the model in equation (5) as derived in[25] relies on ignoring the terms associated with U and $\partial^2 U/\partial x^2$. Since we cannot make statements about the magnitude of these terms, the former omission is only valid provided $p_t \to 0$ or $D_L/D_R \to 1$, while the latter requires $\delta x \to 0$ as well. These relations are in line with observations of the model's behaviour throughout this work.

B Hybrid model derivation

First we consider the case of the membrane placed on the "slow" side, where the diffusion coefficient D_2 is lower. If the walker attempts a crossing from the slow side, it first transits through the membrane with probability P_{b_2} or is reflected with $1-P_{b_2}$. It always transits through the step change in diffusion coefficient, because it enters a region of higher diffusion coefficient. If the walker originates on the fast side, it first needs to pass the step change in diffusion coefficient with probability P_d. Subsequently the membrane on the slow side either permits transit with P_{b_2}, which terminates the interaction, or reflects it with $1-P_{b_2}$, which causes the walker to attempt and always succeed in transiting through the step change in diffusion coefficient. This can be summarised with the two probabilities

$$p_{\text{fast}\to\text{slow}} = P_d P_{b_2}\,, \tag{20a}$$

$$p_{\text{slow}\to\text{fast}} = P_{b_2}\,. \tag{20b}$$

An alternative to the above model is to place the membrane on the "fast" side, where the diffusion coefficient D_1 is higher. This scenario results in walkers reflecting between the two interfaces continuously until eventually exiting on either side. The diagram in figure 10 illustrates this. By summing the probabilities of all different exit conditions, we obtain

$$p_{\text{fast}\to\text{slow}} = \sum_{i=1}^{\infty} P_{b_1}\,(1-P_d)^{i-1}\left(1-P_{b_1}\right)^{i-1} P_d\,, \tag{21a}$$

$$p_{\text{slow}\to\text{fast}} = \sum_{i=1}^{\infty} \left(1-P_{b_1}\right)^{i-1}(1-P_d)^{i-1} P_{b_1}\,. \tag{21b}$$

Here, the infinite sums only converge if $|(1-P_{b_1})(1-P_d)| < 1$, which is the case given that all $P \leq 1$. The probabilities without power of $i-1$ can be removed from the summation and, knowing that

$$\lim_{n\to\infty}\sum_{i=0}^{n}(1-A)^i(1-B)^i = \frac{1}{A+B-AB}\,, \tag{22}$$

we obtain the following expressions for ...

$$p_{\text{fast}\to\text{slow}} = \frac{P_{b_1}P_d}{P_{b_1}+P_d-P_{b_1}P_d}\,, \tag{23a}$$

$$p_{\text{slow}\to\text{fast}} = \frac{P_{b_1}}{P_{b_1}+P_d-P_{b_1}P_d}\,. \tag{23b}$$

To proof that both methods are equivalent we need to show that the following equality is valid

$$P_{b_2} = \frac{P_{b_1}}{P_{b_1} + P_d + P_{b_1} P_d} \tag{24}$$

From[25] we know that

$$P_{b_i} = \frac{\frac{2\sqrt{2\delta t}\kappa}{\sqrt{D_i}}}{1 + \frac{2\sqrt{2\delta t}\kappa}{\sqrt{D_i}}} = \frac{\frac{a}{\sqrt{D_i}}}{1 + \frac{a}{\sqrt{D_i}}} \tag{25}$$

where a is a constant parameter as it only depends on κ and δt. The membrane probabilities from either side (P_{b_1} and P_{b_2}) can then be simplified to

$$P_{b_1} = \frac{ab}{1+ab}, \tag{26a}$$

$$P_{b_2} = \frac{ac}{1+ac}. \tag{26b}$$

where b and c are $\frac{1}{\sqrt{D_1}}$ and $\frac{1}{\sqrt{D_2}}$ respectively. The probability P_D given by Equation (6) can be also rewritten as $P_D = \frac{b}{c}$. If we consider all these simplifications, the right hand side of equation (24) is reduced to the following

$$\frac{P_{b_1}}{P_{b_1} + P_d + P_{b_1} P_d} = \frac{ac}{1+ac} = P_{b_2} \tag{27}$$

validating the equality in equation (24) and showing that both methods give the same probability.

C Flux analysis

Here, we further analyse the steady-state case and offer an explanation for the differences in the reference models' behaviour observed in section 3.2 and elsewhere in this work. Consider the control volume around a barrier bounded by the maximum step length δx on either side as illustrated in figure 11. Note the difference between the running variable x (and its differential dx) and the finite step length δx.

The net flux $J = -D\partial U/\partial x$ can be split into its individual components, i.e. the amount of walkers/concentration crossing from left to right or right to left, whose magnitudes are given by:

$$J_{L\to R} = \frac{1}{\delta t}\int_{-\delta x_L}^{0} + \frac{U(x)}{2} p_{t,L\to R}(x)\,\mathrm{d}x, \tag{28a}$$

$$J_{R\to L} = \frac{1}{\delta t}\int_{0}^{\delta x_R} - \frac{U(x)}{2} p_{t,R\to L}(x)\,\mathrm{d}x. \tag{28b}$$

The integral bounds on the two sides cover the farthest that a walker can be located away from the boundary on either side in order for its step δx to interact with the barrier. While the concentration density $U(x)$ in general depends on position x, we ignore this dependency here because we assume $U =$ constant in the domain for the steady-state case. The factor $^1/_2$ accounts for the fact that only half the walkers are expected to step towards the barrier. The $\pm$ signs are included to designate the directionality of the flux components J such that $\sum J = 0$. For simplicity, we now omit this and use *magnitudes* only. We also cancel the terms U and δt and henceforth use the probability integrals F:

$$F_{A\to B} = \int p_{t,A\to B}(x)\,\mathrm{d}x. \tag{29}$$

The steady-state solution requires the net flux $\sum J = 0$, i.e. $F_{L\to R} = F_{R\to L}$. If not, an imbalance in $U(x)$ will develop. If the diffusivities on both sides are equal ($D_L = D_R = D$), the models considered in this work reduce to $p_{t,L\to R} = p_{t,R\to L}$. Furthermore, by definition $\delta x_L = \delta x_R$ and hence $\sum J = 0$. Next, we consider the different models for the non-trivial case $D_L \neq D_R$.

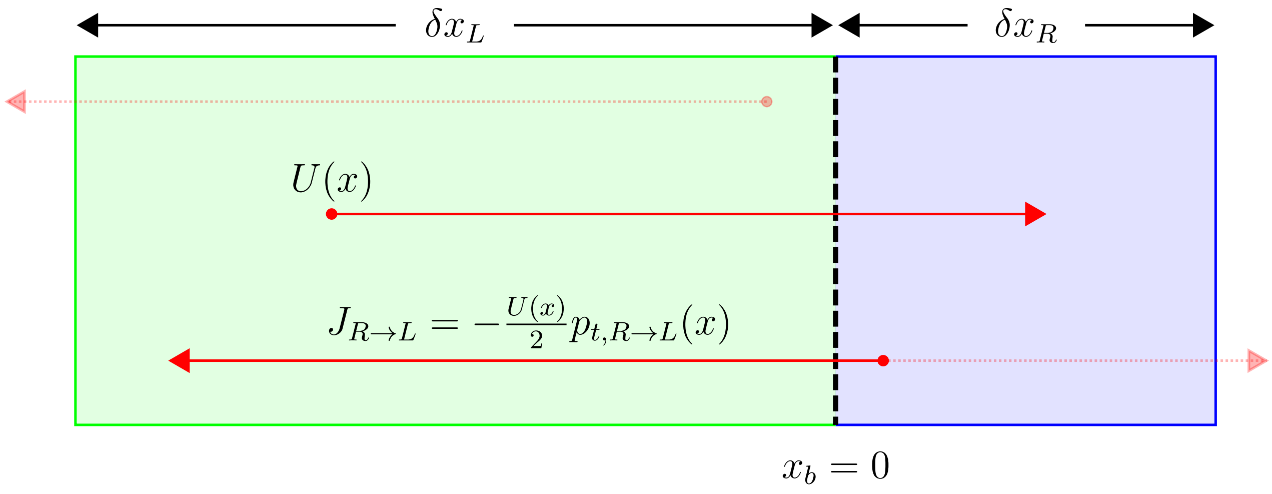

Figure 11. A graphical explanation of the flux analysis around two compartments ($D_L > D_R$) separated by a permeable barrier with asymmetric transit probabilities $p_{t,L\to R}$ and $p_{t,R\to L}$. The control volume is bounded by the step lengths δx on either side. Walkers located inside this volume at a point with concentration/density $U(x)$ add to the flux component J with their weighted contribution $\pm U(x) p_t(x)$, provided they step towards the barrier (accounted for by the factor $^1/_2$).

C.1 Interface model

In the case of an infinitely permeable membrane, we consider the interface model[29]. The probability of transit for interfaces with discontinuous diffusivity D (ignoring permeability κ) is given by

$$p_{t,L\to R} = \min\left(1, \sqrt{\frac{D_R}{D_L}}\right), \tag{30a}$$

$$p_{t,R\to L} = \min\left(1, \sqrt{\frac{D_L}{D_R}}\right). \tag{30b}$$

For this model, the probabilities p_t do not depend on x but only on D. As a result, we can remove p_t from the integral and simplify the result:

$$F_{L\to R} = p_{t,L\to R} \int_{-\delta x_L}^{0} \mathrm{d}x = p_{t,L\to R}\,\delta x_L = p_{t,L\to R}\sqrt{2 D_L \delta t}\,, \tag{31a}$$

$$F_{R\to L} = p_{t,R\to L} \int_{0}^{\delta x_R} \mathrm{d}x = p_{t,R\to L}\,\delta x_R = p_{t,R\to L}\sqrt{2 D_R \delta t}\,. \tag{31b}$$

To remove the awkward min-operator for p_t, consider the two distinct cases in table 1 separately. For both combinations of p_t values it is clear that $F_{L\to R} = F_{R\to L}$. In fact, $F = \sqrt{2 D_{\min} \delta t}$ which we also observe in numerical simulations with $\kappa = \infty$.

Table 1. The two cases of D_L and D_R considered in equation (30) and their corresponding probabilities of transit p_t and resulting fluxes F.

D	$p_{t,L\to R}$	$p_{t,R\to L}$	F
$D_L > D_R$	$\sqrt{\frac{D_R}{D_L}}$	1	$\sqrt{2 D_R \delta t}$
$D_L < D_R$	1	$\sqrt{\frac{D_L}{D_R}}$	$\sqrt{2 D_L \delta t}$

C.2 Membrane model

The authors in[25] introduce two probabilities of transit p_t, going from L to R and from R to L:

$$p_{t,L\to R} = \frac{2\kappa s_L}{D_L + 2\kappa s_L} \approx \frac{2\kappa s_L}{D_L}\,, \tag{32a}$$

$$p_{t,R\to L} = \frac{2\kappa s_R}{D_R + 2\kappa s_R} \approx \frac{2\kappa s_R}{D_R}\,. \tag{32b}$$

Each p_t depends on the diffusivity D of the original compartment and the distance s from the step origin to the barrier. The latter is equal to $\mp x$ since $x_b = 0$, such that $s_L \in [-\delta x_L, 0]$ and $s_R \in [0, \delta x_R]$ respectively. Note that the simplification on the right-hand sides of equation (32) only applies if $2\kappa s \ll D$. We will analyse both cases below.

C.2.1 Larger time step

Using the full, non-approximated term for p_t from equation (32), we obtain the following:

$$F_{L\to R} = \int_{-\delta x_L}^{0} p_{t,L\to R}(x)\,\mathrm{d}x = \int_{-\delta x_L}^{0} \frac{x}{x - \frac{D_L}{2\kappa}}\,\mathrm{d}x\,, \tag{33a}$$

$$F_{R\to L} = \int_{0}^{\delta x_R} p_{t,R\to L}(x)\,\mathrm{d}x = \int_{0}^{\delta x_R} \frac{x}{x + \frac{D_R}{2\kappa}}\,\mathrm{d}x\,. \tag{33b}$$

The integrals can be solved to give:

$$F_{L\to R} = \delta x_L + \frac{D_L}{2\kappa} \ln\left(\frac{D_L/2\kappa}{\delta x_L + D_L/2\kappa}\right)\,, \tag{34a}$$

$$F_{R\to L} = \delta x_R + \frac{D_R}{2\kappa} \ln\left(\frac{D_R/2\kappa}{\delta x_R + D_R/2\kappa}\right)\,. \tag{34b}$$

Since $F_{L\to R}$ and $F_{R\to L}$ depend on D_L and D_R respectively, and because F cannot be reduced further, we conclude that $F_{L\to R} \neq F_{R\to L}$ and thus $\sum J \neq 0$ as observed in section 3.

C.2.2 Sufficiently small time step

For a sufficiently small time step δt, we can solve for the simplified expression of p_t:

$$F_{L\to R} = \int_{-\delta x_L}^{0} p_{t,L\to R}(x)\,\mathrm{d}x = \frac{2\kappa}{D_L}\int_{-\delta x_L}^{0} -x\,\mathrm{d}x = \frac{\kappa \delta x_L^2}{D_L} = 2\kappa\delta t\,, \tag{35a}$$

$$F_{R\to L} = \int_{0}^{\delta x_R} p_{t,R\to L}(x)\,\mathrm{d}x = \frac{2\kappa}{D_R}\int_{0}^{\delta x_R} x\,\mathrm{d}x = \frac{\kappa \delta x_R^2}{D_R} = 2\kappa\delta t\,. \tag{35b}$$

Hence, $F_{L\to R} = F_{R\to L} = 2\kappa\delta t$ (we confirm this in simulations with small δt) and the model will retain the steady-state solution. Recall that the simplification above requires that $2\kappa\delta s \ll D$. This can be recast to

$$1 \ll \frac{2\kappa\sqrt{2D\delta t}}{D} = \sqrt{\frac{8\kappa^2\delta t}{D}} \tag{36}$$

and we now observe that the threshold at which a significant error in the fluxes occurs increases with κ and δt and decreases with D. This is in line with observations in figure 5.

C.3 Asymmetric interface reflection

As originally stated in[11], the interface reflection condition must be respected even as $\kappa \to \infty$. This is satisfied intrinsically in equation (30). Following from equation (32), the ratio of probabilities

$$\frac{p_{t,L\to R}}{p_{t,R\to L}} = \frac{2\kappa\delta x_{i,L}}{2\kappa\delta x_{i,R}} \frac{D_R + 2\kappa\delta x_{i,R}}{D_L + 2\kappa\delta x_{i,L}} \tag{37}$$

approaches unity in the limit of $\kappa \to \infty$ instead of $\sqrt{D_R/D_L}$. This might explain why the model appears to break down as the time step restriction is exceeded either by increasing δt or κ as done in figure 5.

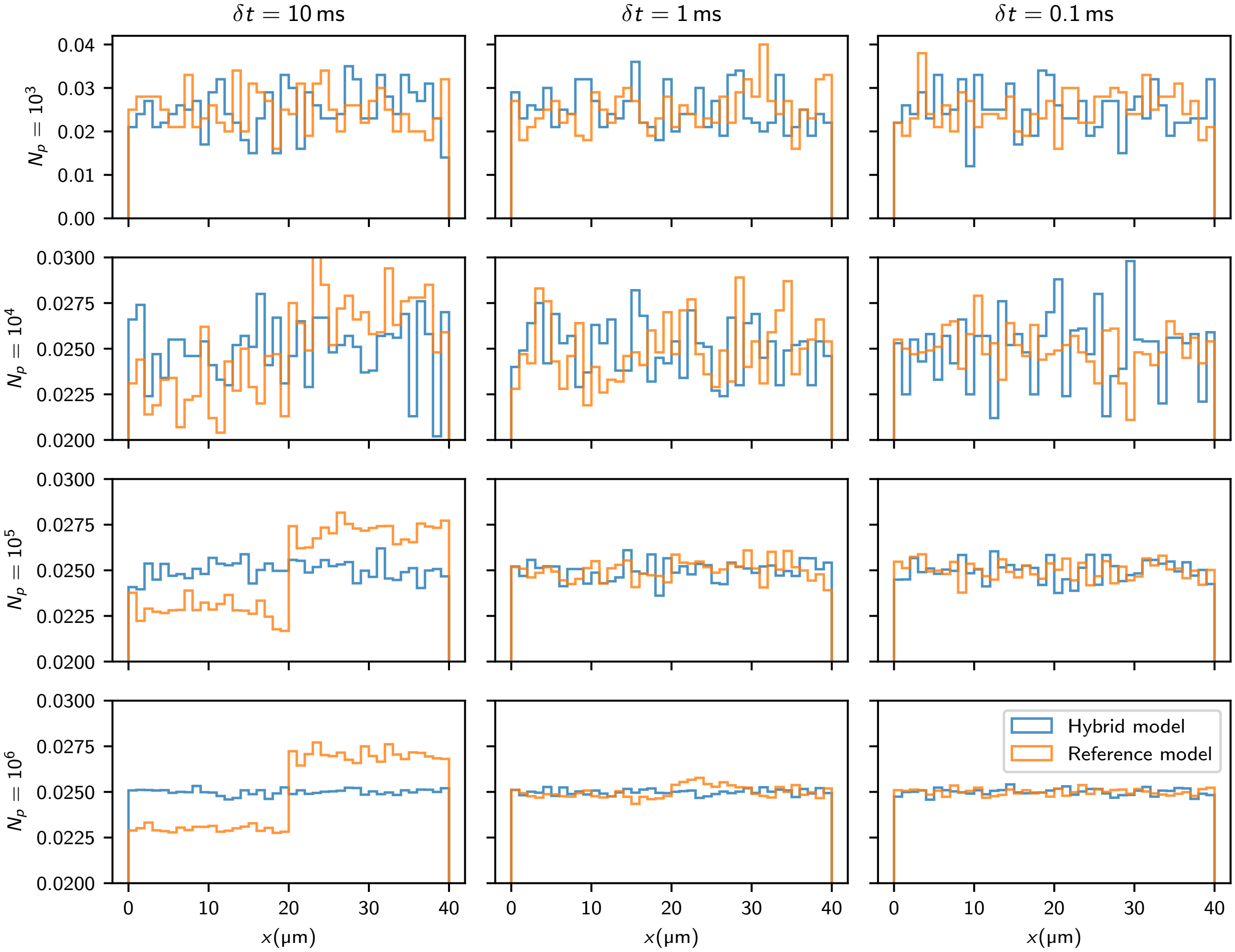

Figure 12. Convergence of random walk simulations of the steady-state in figure 3 using both transit models for varying number of walkers N_p and time steps δt. The total simulated time is $t = 100\,\text{ms}$ and walkers are initially seeded uniformly in the domain ($D_L = 2.5\,\mu\text{m}^2/\text{ms}$, $D_R = 0.5\,\mu\text{m}^2/\text{ms}$, $\kappa = 0.05\,\mu\text{m}/\text{ms}$).

D Convergence

Figure 12 shows the histograms of walker positions for both transit models considered in this work, equations (5) and (6). We consider three time steps and increase the number of walkers until the random fluctuations are of an acceptable level (compare section 3.2).

.
